# The widening medication adherence gap

**DOI:** 10.1371/journal.pmed.1005237

**Published:** 2026-09-04

**Authors:** Zachary A. Marcum

**Affiliations:** Department of Pharmacy, School of Pharmacy, University of Washington, Seattle, Washington, United States of America

## Abstract

In this Perspective, Zachary Marcum argues that the gap between medication efficacy in trials and real-world effectiveness is widening, and with it the opportunity cost of medication nonadherence. He discusses why closing this gap requires not just individual behavior change, but system-level solutions.

## Teaser

The gap between medication efficacy in trials and real-world effectiveness is widening, and with it the opportunity cost of medication nonadherence. Closing this gap requires more than individual behavior change; it requires system-level solutions, including coverage and funding design, pharmacist-led programs, and health information technology.

Recent years have seen the development of medications that produce clinically meaningful weight loss [1], anticoagulants that prevent stroke with minimal monitoring [2], and vaccines that may reduce the risk of dementia [3,4], among other therapeutic advances. Yet across therapeutic areas, a substantial proportion of patients discontinue, underuse, or never fill these prescriptions. This issue has persisted for decades [5], but the consequences have never been greater. The medications at stake are more effective than those of a generation ago [1,2,6]; I argue that the widening gap between the efficacy demonstrated in trials and the effectiveness observed in practice is driven less by pharmacology than by medication nonadherence; a problem shaped not only by individual behavior but by the systems within which patients seek care.

Nowhere is this gap more visible than with the use of glucagon-like peptide-1 receptor agonists (GLP-1 RAs) for weight loss management. A recent synthesis of real-world evidence reveals that 20%–50% of patients discontinue GLP-1 RAs within the first year, many use substantially lower doses than those studied in trials, and the weight loss observed in clinical practice is consistently lower than in randomized controlled trials; outcomes approach trial results among highly adherent patients, however [7]. The drivers of early discontinuation—gastrointestinal side effects, cost, insurance barriers, and supply shortages—are familiar, but the clinical consequences of stopping are not uniform. For patients prescribed GLP-1 RAs solely for weight management, discontinuation results primarily in weight regain. But GLP-1 RAs also reduce the risk of cardiovascular events and clinically important kidney disease outcomes compared with placebo [8]. Because these benefits depend on continued exposure rather than conferring durable protection after withdrawal, discontinuation in such patients forfeits not only weight loss but reductions in morbidity and mortality.

Medication adherence is also an issue for anticoagulants; a systematic review and meta-analysis of nearly 595,000 patients with atrial fibrillation found that only two-thirds maintained good adherence to direct oral anticoagulants, and nonadherence was associated with a 39% increase in the risk of stroke [9]. This finding quantifies the gap from prescribed therapy to delivered benefit even for medications whose value is unambiguous and whose dosing is straightforward, reframing nonadherence from a behavioral concern into a measurable contributor to preventable morbidity and mortality.

A different kind of adherence shortfall is playing out with the recombinant zoster vaccine (RZV), which protects against herpes zoster (shingles). RZV requires only two doses, separated by 2–6 months. Yet a nationwide study of more than 726,000 US adults found that adherence to the recommended schedule was only 72%, with completion rates markedly lower among racial and ethnic minorities, younger adults, and those with lower household income [10]. Emerging evidence suggests that what is at stake may be far greater. A large-scale longitudinal analysis of US health records found that herpes zoster vaccination was associated with a reduced risk of dementia, with stronger protection in those who received both doses of the recombinant vaccine compared with a single dose [3]. A target trial emulation among nearly 510,000 skilled-nursing facility residents similarly found that RZV was associated with a reduction in dementia risk [4]. If these findings are confirmed [11], every person who fails to complete the series may be forgoing not only protection against a painful dermatologic condition but also a potential modification of dementia risk.

What is striking is not the magnitude of the underlying problem, which has changed little in decades [5]. Rather, I argue that the opportunity cost of medication nonadherence—the benefit forgone when effective therapy goes untaken—deserves renewed attention. This claim cannot be directly tested, but its logic is straightforward: when adherence rates are static and treatment effects grow, the benefit forgone through nonadherence grows. Clinical trials for contemporary medications may demonstrate efficacy exceeding that of previous generations [1,2,6]. In a network meta-analysis, for example, semaglutide reduces bodyweight by 11.4% from baseline compared to 3.1% for orlistat [1]; similarly, direct oral anticoagulants reduce stroke or systemic embolism by 19% compared to warfarin [2], and RZV reduces zoster risk by 92% versus 51% for its live attenuated predecessor [6]. But these benefits are realized only under the conditions of trial participation. In practice, where patients bear cost-sharing and manage competing demands, the attenuation of benefit is substantial. It is, however, important to acknowledge that improvements in subsequent therapies or in population health could offset some of this loss in therapeutic benefit in particular settings.

Moreover, with the introduction of therapies costing tens of thousands of dollars per year, improving adherence can increase short-term expenditures (a tension for payers), though the downstream costs of preventable hospitalizations and disease progression are far greater. Nonadherence itself is heterogeneous—ranging from cost-related gaps in treatment to unintentional lapses in complex regimens—and effective solutions must account for this variation. Nor is all medication discontinuation actually nonadherence. Stopping a therapy after an informed discussion of adverse effects with a healthcare provider is shared decision-making, and it is important not to conflate these when conducting research and designing solutions.

Established interventions, including patient education, simpler dosing schedules, and improved communication, remain necessary but insufficient. At the bedside, treating adherence as a clinical decision point means counseling on the cardiorenal benefits at risk when a GLP-1 RA is stopped, checking persistence at every anticoagulant renewal, and counseling about the importance of completing the RZV series. But individual effort has limits. In fact, a Cochrane review of 182 randomized trials found that even the most effective adherence interventions, typically bundles of education, counseling, and ongoing support from health system professionals, produced only modest improvements in adherence or clinical outcomes [12].

More broadly, what is needed is a system-level reconceptualization of adherence as a determinant of therapeutic value. By system-level, I mean interventions that change the environment in which prescriptions are written and filled, and medications are consumed. For GLP-1 RAs, this means addressing the structural barriers—cost, insurance coverage, and supply—that drive discontinuation. For chronic medications, it means expanding pharmacists’ authority to manage therapy through collaborative practice agreements and transition-of-care programs, and aligning cost-sharing with therapeutic value through value-based insurance design. For multi-dose vaccines, it means integrating completion tracking into electronic health records. An example of a successful system-level intervention is a randomized trial of nearly 6,000 patients after myocardial infarction that eliminated copayments for cardiovascular medications, improving adherence by 4%–6% and reducing rates of first major vascular events without increasing total health spending [13]. System-level barriers, and thus solutions, may take different forms across health systems, but the principle holds.

Compounding these challenges, nonadherence is difficult to detect in routine practice; clinicians consistently underestimate it, and available measures, such as pharmacy refill records, self-report, and electronic health record documentation, each capture only a partial picture. Artificial intelligence may eventually help close this gap, though rigorous evidence that these tools improve clinically meaningful endpoints remains limited [14].

The limiting factor in realizing the full value of modern therapeutics is no longer only pharmacology; it is also human behavior, reinforced by systems that make adherence difficult. Until we invest in solving this problem with the same rigor we apply to drug discovery—through coverage and funding design, pharmacist-led programs, and health information technology, coupled with implementation research—we will continue to pay for the promise of transformative therapies while realizing only a fraction of their benefit.
